# Poor awareness of stroke educational tools among older adults in China

**DOI:** 10.1002/brb3.2357

**Published:** 2021-09-14

**Authors:** Ling Ling, Zhongcheng Li, Sichen Yao, Xiaochuan Liu, Jing Zhao

**Affiliations:** ^1^ Department of General Medicine Qibao Community Health Service Center Shanghai China; ^2^ Department of General Medicine Wujing Community Health Service Center Shanghai China; ^3^ WanNan Medical College Anhui China; ^4^ Department of Neurology, Minhang hospital Fudan university Shanghai China

**Keywords:** awareness, community resident, FAST, older adult, Stroke 1‐2‐0, stroke

## Abstract

**Background:**

Stroke 1‐2‐0 and FAST (Face, Arm, Speech, Time) are two popular stroke educational tools that have been used in many stroke promotion campaigns. However, few researchers have investigated awareness of these tools among older adults in communities.

**Methods:**

This study was a cross‐sectional survey of community‐living older adults. Two family physicians conducted face‐to‐face interviews with older adults living in Minhang district, Shanghai, between October 1, 2020 and November 31, 2020. The survey comprised three parts: basic information, prior medical history, and stroke awareness knowledge. We focused on the awareness of FAST and Stroke 1‐2‐0 and investigated factors associated with awareness of these stroke educational tools.

**Results:**

The sample of this study was 466 older adults. Their mean age was 73.45 years. Male respondents accounted for 46.14% of the total sample. More than half of the older adults surveyed had an educational background of less than 6 years. Over 90% of the older adults surveyed had never heard about Stroke 1‐2‐0 or FAST. The awareness rate of Stroke 1‐2‐0 and FAST was 7.94%, with awareness of Stroke 1‐2‐0 being higher than that of FAST (6.01% vs. 0.43%, *p* < .05). None of the respondents who had heard about the two stroke educational tools could explain the utility of either tool fully. Having a background in higher education was associated with awareness of stroke educational tools independently, with an odds ratio (OR) of 10.07, 95% confidence interval (CI) of 3.7–27.4, *p* < .001. In addition, Wechat (OR 6.57, 95%CI 2.65–16.27, *p* < .001) and the community bulletin board (OR 2.95, 95%CI 1.37–6.33, *p* = .005) were found to be important sources for acquiring knowledge of stroke awareness tools.

**Conclusion:**

The limited awareness of Stroke 1‐2‐0 and FAST displayed among older adults in the community indicates that we must take action to improve education on stroke among the elderly.

## 1 INTRODUCTION

Stroke is the leading cause of death and disability among adults worldwide (Feigin et al., 2014; Wu et al., 2019 ). In China, there are approximately 250 million new onset stroke cases each year, a situation that is expected to worsen considering the rapid growth of China's aging population (Wang et al., 2020). Since acute stage therapies for ischemic stroke, such as intravenous thrombolytic therapy, are time dependent, individuals must act properly and immediately once a stroke has been identified. The typical prehospital delay for a Chinese ischemic stroke patient is devastatingly long according to a multicenter, hospital‐based study that indicated the median prehospital delay is 15 h (Jin et al., 2012). Yet more shocking is the fact that only 12.5% of ischemic stroke patients were transported to hospital by emergency medical services (EMS) according to data from the Chinese Stroke Center Alliance (Gu et al., 2019)

Raising public awareness of stroke has always been seen as a promising strategy for improving public response to stroke. Several stroke awareness tools for educating the public on stroke symptoms, including FAST (Face, Arm, Speech, Time) (Kleindorfer et al., 2007), Stroke 1‐2‐0 (Zhao & Liu, 2017 ), and Stroke 112 (Zhao et al., 2018), have been promoted. Our previous research indicated that Stroke 1‐2‐0 is better accepted than FAST as a public stroke educational tool among the Chinese population (Zhao et al., 2020). Despite the fact that the Stroke 1‐2‐0 educational campaign was launched 2 years ago, the rate at which such stroke educational tools increase public awareness remains unknown. Thus, we set out to investigate awareness of Stroke 1‐2‐0 and FAST among community‐living residents, focusing on older adults because limited research has targeted this group specifically. We also investigated factors associated with awareness of stroke educational tools.

## 2 METHODS

### 2.1 Study design and population

This study was a cross‐sectional survey conducted among community‐living older adults. Two family physicians and members of their medical team conducted face‐to‐face interviews with the older adults living in two suburban communities in Minhang district, Shanghai, between October 1, 2020 and November 31, 2020. These two communities were randomly selected out of all the communities in the suburban area of Minhang district. The survey was conducted when community residents came into the community health center to seek medical advice on the management of chronic diseases such as hypertension or diabetes. Our sample was community residents over the age of 60 years who were free of any acute stage cardiovascular diseases. We only invited one member of each household who was eligible to complete this survey. We excluded from our data all the residents who refused to participate after understanding the purpose of the study.

The survey consisted of three parts: basic information, prior medical history, and stroke awareness knowledge. Details of the survey are available in the online Supplementary Material. Briefly, participants were asked questions related to the awareness of stroke symptoms and risk factors, related to the actions they would take if they spotted a stroke, and related to their knowledge of stroke awareness tools. For example, interviewers asked participants whether they would think someone was having a stroke if they suddenly complained of having trouble seeing out of one or both eyes. Participants who responded with the answer “no” or “I don't know” were categorized as not having knowledge of the symptoms of stroke. For measuring knowledge of stroke awareness tools, we included two popular and widely used stroke educational tools: Stroke 1‐2‐0 and FAST. Participants were asked whether they had ever heard about them before and whether they could explain the meanings of the numbers or letters included in these tools. Respondents were then categorized into three groups: those who “know all the meanings,” those who “know part of the meanings,” and those who “don't know any of it.” A good level of stroke educational tool awareness was defined as having heard about Stroke 1‐2‐0 or FAST.

### 2.2 Data collection

About 515 households with family members over the age of 60 living in them were identified in the two communities. We were required to interview older adults from at least 464 of these households in order to ensure 90% coverage of the population. The family physicians and their team members were trained by stroke physicians before conducting the face‐to‐face interviews to make sure there were no inconsistencies in the interview script used in the survey. Prior medical history was defined according to standard definitions and the medical records of each participant we checked.

### 2.3 Sample size calculation

Based on our prior work, the awareness of Stroke 1‐2‐0 and FAST was 50.1% and 19.1% among 3066 participants in 2016 (Zhao et al., 2020). However, more than 90% of the responders included in that study were aged below 60 and the awareness of stroke education tools varied much among participants living in different areas across China. In consideration of these differences, we consider it is reasonable to set the awareness rate of stroke education tools (Stroke 1‐2‐0 and FAST) at 10–20% to produce a two‐sided 95% confidence interval. We applied these two proportions in PASS 15.0.5 software (Fleiss et al., 2003, Newcombe, 1998), which indicates the sample size should range from 407 to 914.

### 2.4 Statistical analysis

The entire statistical analysis was performed on SPSS version 25.0 (IBM Corp., New York). We analyzed categorical variables using the chi‐square test or the Fisher exact test. As for continuous variables, we applied a two‐sample *t*‐test. Factors associated with the awareness of stroke education tools were investigated by using logistic regression analysis. Baseline characteristics (age, gender, educational background, smoking history, drinking habit, hypertension, diabetes, myocardial infarction, prior stroke, dyslipidemia, cancer history, atrial fibrillation) with *p* values less than .05 were adjusted in the multivariate logistic regression analysis to target variables that are independently associated with the awareness of stroke education tools. Due to the low proportion of older adults with college degrees who took part in this study, we converted educational background into a binary variable (primary school education vs. above primary school education) in the logistic regression. We also translated smoking history and drinking habit into binary variables by treating both participants who had quit and those who had never smoked as nonsmokers. A forest plot was drawn in R version 3.6.1 (The R Foundation for Statistical Computing). A two‐tailed *p* value < .05 was considered to have statistical significance.

## 3 RESULTS

### 3.1 Population characteristics

A total of 511 older adults were invited to participate in the survey and the response rate was 91.19% (15 refused to participate, 20 were not able to complete the survey by themselves and were accompanied by their children, and 10 decided to drop out in the middle of the survey). Finally, 466 elderly persons were included in this study. No missing information was found in all surveys. Table 1 shows the baseline characteristics of all the residents. The mean age of the study population was 73.45 years. Male respondents accounted for 46.14%. Most of them had an educational background of less than 6 years. The proportion of respondents who continued smoking and drinking were 13.52% and 8.8%, respectively. More than half of respondents reported that they had never drunk or smoked in their entire life. Regarding medical history, hypertension (65.24%) was the most common chronic disease among respondents, followed by dyslipidemia (33.05%) and diabetes (24.89%).

**TABLE 1:** Characteristics of the study population

	*N* = 466
	Mean, Standard Deviation/N, %
Age	73.45(6.88)
Gender(male)	215(46.14%)
Education background	
Primary school	250(53.65%)
Secondary school	181(38.84%)
High school	33(7.08%)
College degree	2(0.43%)
Smoking habit	
Quit	88(18.88%)
Never quit	63(13.52%)
Never smoke	315(67.60%)
Alcohol habit	
Quit	115(24.68%)
Never quit	41(8.80%)
Never drink	310(66.52%)
Medical history	
Hypertension	304(65.24%)
Diabetes	116(24.89%)
Prior MI	16(3.43%)
Prior stroke	30(6.44%)
Dyslipidemia	154(33.05%)
AF	7(1.5%)
Cancer history	9(1.93%)

Abbreviations: MI, myocardial infarction; AF, atrial fibrillation.

### 3.2 Awareness of stroke‐related knowledge

**FIGURE 1:**
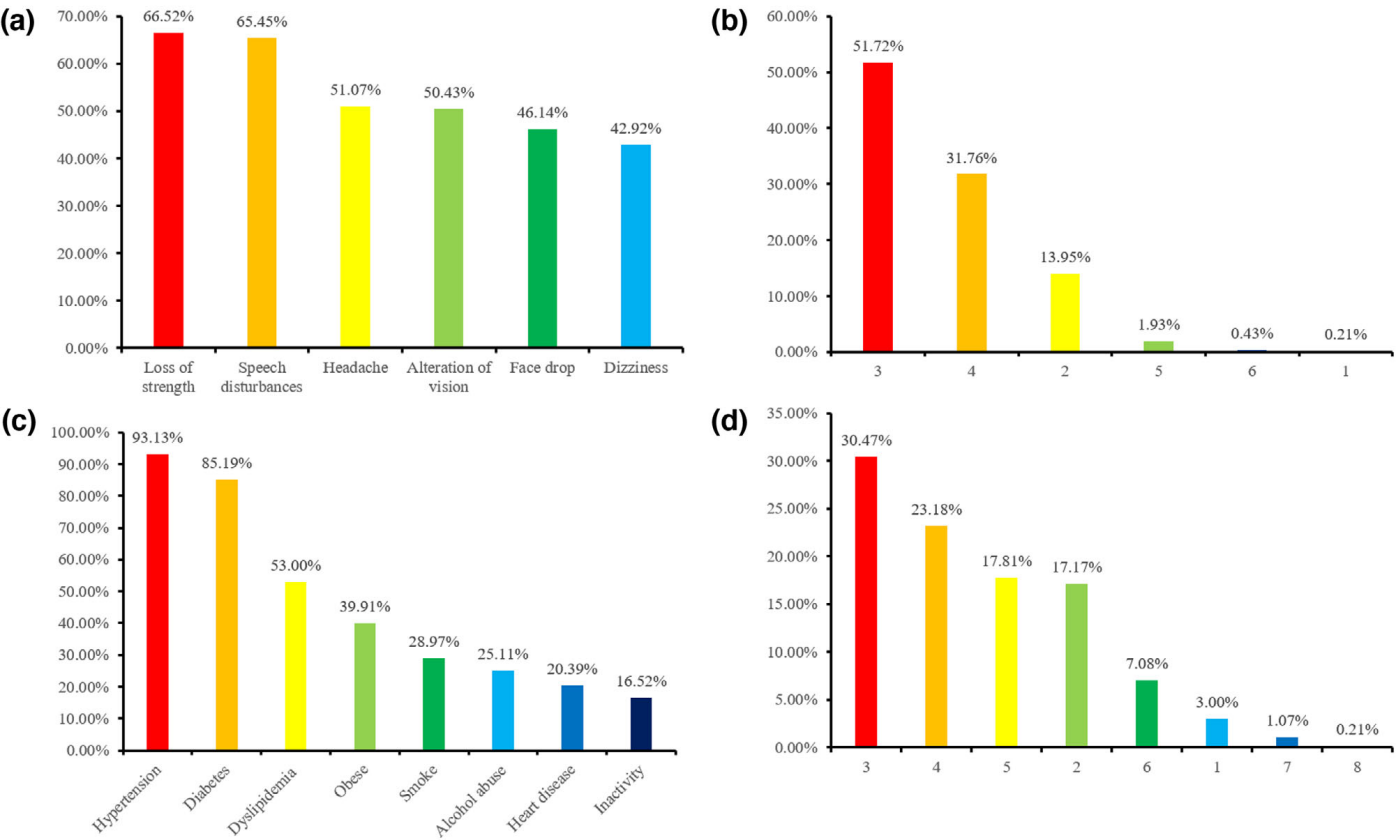
The awareness of stroke symptoms and risk factors in community older adults. (a) The awareness of six stroke symptoms. (b) The awareness of the number of stroke symptoms. (c) The awareness of eight stroke risk factors. (d) The awareness of the number of stroke risk factors

Figure 1 shows participants’ awareness rate of six common stroke symptoms and eight risk factors among community‐living older adults. The symptom of sudden weakness in the arm or leg (66.52%) was the most well known of all stroke symptoms, whereas sudden dizziness was found to be the least known stroke symptom (42.92%). It is shocking to note that no participant reached an awareness rate of 80%. Most of them (51.72%) knew only three out of the six common stroke symptoms. As for the eight stroke risk factors, hypertension (93.13%) and diabetes (85.19%) were the most well known. The importance of regular exercise and not being static all day for preventing stroke was only known to 16.52% of respondents. To our surprise, less than 40% of respondents were able to name multiple stroke risk factors in the interview.

**FIGURE 2:**
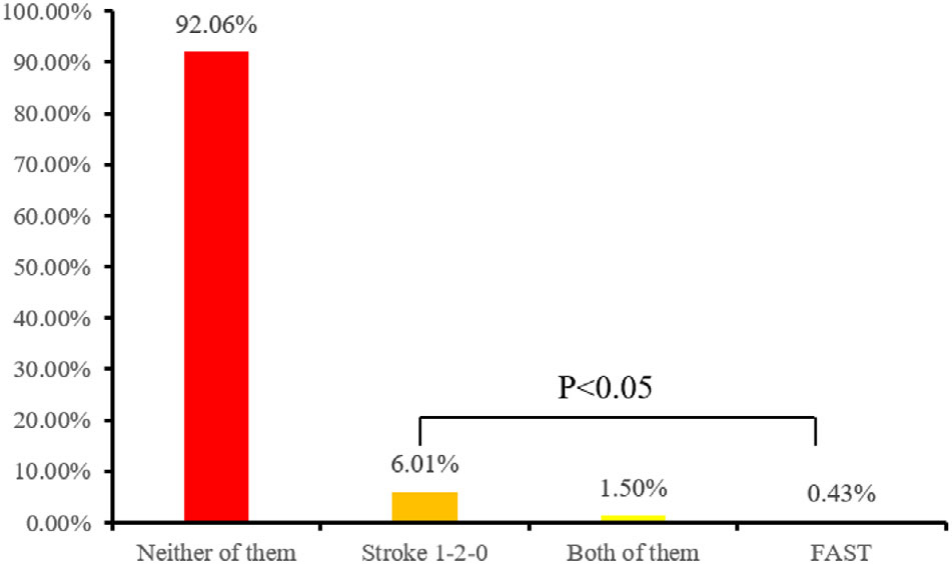
The awareness of stroke educational tools

Figure 2 shows participants’ awareness of stroke educational tools. Over 90% of respondents had never heard about Stroke 1‐2‐0 and FAST. The awareness rate of Stroke 1‐2‐0 and FAST was 7.94%, and Stroke 1‐2‐0 was more familiar to respondents than FAST (6.01% vs. 0.43%, *p* < .05). Only 1.5% of them had knowledge of both. Finally, none of the 37 respondents who had heard about Stroke 1‐2‐0 and FAST could explain the full significance of the awareness tools. Just five (55.56%) of the nine residents who had heard about FAST were capable of partially explaining the meaning of FAST, whereas 27 (77.14%) of the 35 residents who had heard about Stroke 1‐2‐0 could partially explain the meaning of Stroke 1‐2‐0.

Regarding knowledge of acute stage therapy for ischemic stroke, only 10.09% of respondents had heard about intravenous thrombolytic therapy before and nearly 90% of respondents had never heard of any of them. Knowledge of thrombectomy therapy was significantly lower than knowledge of intravenous thrombolytic therapy (0.64% vs. 10.09%, *p* < .05). It is surprising to note that 6.01% of participants responded that they would not call EMS when they realized they were having a stroke. The common reasons for this were that they would want to call their children to ask for their advice, the EMS is expensive, and the EMS is slow.

**FIGURE 3:**
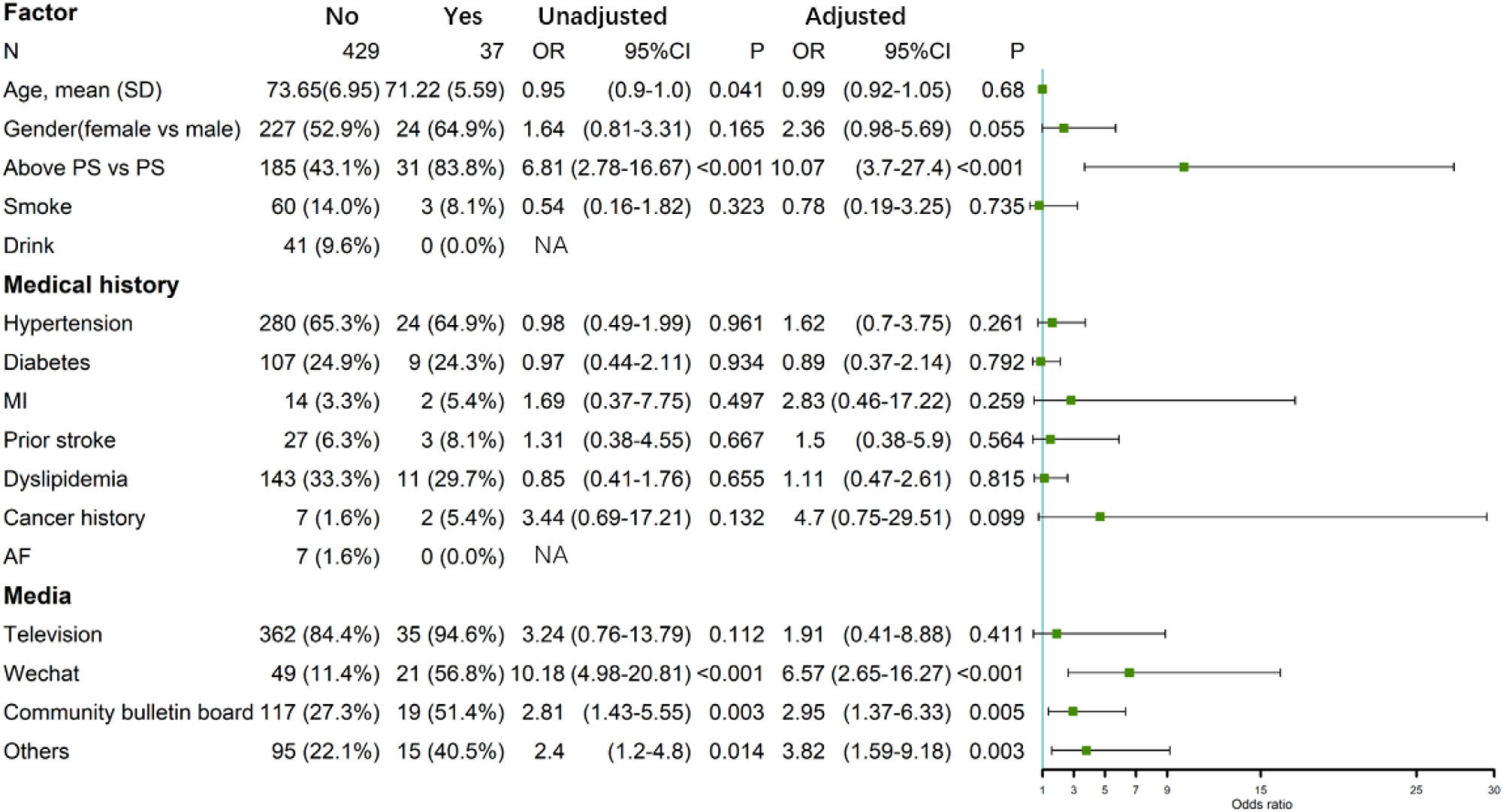
Factors associated with the awareness of stroke educational tools, Abbreviation: PS, primary school; MI, myocardial infarction; AF, atrial fibrillation; NA, not appliable; OR, odds ratio; CI, confidence interval

The most common source for acquiring stroke‐related knowledge was identified as television (85.19%). Community bulletin boards (29.18%) were identified as another important way of acquiring health knowledge. Figure 3 shows the factors associated with awareness of stroke educational tools (including Stroke 1‐2‐0 and FAST). Younger age and a background in higher education were significantly associated with knowledge of stroke awareness tools in the univariate analysis. However, age was not an independent variable in the multivariate analysis (*p* = .68). The awareness of stroke educational tools was higher in women in the multivariate analysis, although it did not reach statistical significance (odds ratio (OR) 2.36 95% and CI 0.98–5.69, *p* = .055). Although television was reported as the most common source for acquiring health knowledge, it was not related to the awareness of stroke educational tools (Figure 3). On the contrary, Wechat (OR 6.57, 95%CI 2.65–16.27, *p* < .001), community bulletin boards (OR 2.95, 95%CI 1.37–6.33, *p* = .005), and other sources (OR 3.82, 95%CI 1.59–9.18, *p* = .003) were found to be independently associated with knowledge of stroke awareness tools.

## 4 DISCUSSION

Our study indicated that not only are there poor levels of stroke awareness among community‐living older adults, but also that there is poor awareness of stroke educational tools. The current study adds value to previously published research, highlighting the fact that attention should also be given to promoting stroke educational tools as well as educating older people on common stroke symptoms and risk factors. Stroke educational tools are promising in that they promote awareness of stroke symptoms for people without a medical background and they have been applied in many stroke educational campaigns. However, our study showed that a person's educational background plays a critical role in their awareness of stroke educational tools. Thus, our results suggest that in the future, we should expend more effort to raising awareness among older adults who do not have a strong educational background, such as those with primary school education and below.

Consistent with a previous community‐based questionnaire (Sun et al., 2011), we found that the most well‐known stroke symptom was weakness of the arm or leg, whereas hypertension was identified as the most common stroke risk factor. This previous survey included 2519 participants from four cities (Beijing, Shanghai, Changsha, and Chengdu) in China. They also found that awareness of other stroke symptoms apart from that of weakness of the arm or leg ranged from 58.2% to 71.2%, a result that is higher than our own. The reason for this may be that they included a younger population in their study (with a mean age of 55.72) and that they included participants with a stronger educational background (more than half of them had finished primary school). In this study, they also found the lead source for acquiring stroke‐related knowledge to be television, mirroring our study.

Another study conducted in Chongqing, China, revealed the same phenomenon, indicating low levels of awareness of stroke symptoms and risk factors among community residents (Yang et al., 2014). They also discovered that only 23.3% of participants were aware of thrombolytic therapy in the same population (Yang et al., 2014). The differences between their study and our own regarding awareness of thrombolytic therapy may have been caused by the sociodemographic disparity. Their study did not target members of the older population who were more vulnerable to stroke, especially within the context of global aging. Bhat et al. (2021) found low awareness of stroke symptoms and risk factors among elderly hypertensives in India in a study that indicated that 40% of them had never heard the term “stroke.”

One study in Argentina (Caruso et al., 2015) found only 65% of the 367 elderly adults surveyed would call EMS, even when they were experiencing typical stroke symptoms. Although the results of our study indicate that 93.99% of respondents would call EMS when they experienced symptoms of stroke, our results should be interpreted with caution, as in most scenarios, it is quite difficult for community residents to correctly identify the onset of stroke. Some may choose to rest for a few hours to see if their symptoms disappear. Among the respondents who indicated they would refuse to call EMS when they experienced symptoms of stroke, the most common reason for their refusal was that they felt they would need to ask for their children's advice. This is quite a common and undesirable phenomenon, as older adults tend to stay home alone while their children work in the city center.

Stroke 1‐2‐0 and FAST are two popular stroke educational tools. FAST has been widely used in many stroke educational campaigns in Western countries (Bray et al., 2013; Dombrowski et al., 2013; Hartigan et al., 2014; Nordanstig et al., 2019 ). Some of these countries translated FAST, creating their own acronym in order to eliminate the language barrier from preventing understanding of the signification of each letter. In 2016, a novel version of FAST called Stroke 1‐2‐0 was created by Chinese researchers (Zhao & Liu, 2017; Zhao & Liu, 2017 ). It embedded three stroke symptoms into the Chinese EMS number system of 120, which means: 1, check for uneven face; 2, check for weakness in the arm; and 0, check for absence of clear speech. To date, it has been shown that this tool can improve stroke knowledge among community physicians (Liu et al., 2019) and middle school students (Li et al., 2020). A nationwide online survey that gathered 20,000 respondents indicated that public acceptance of Stroke 1‐2‐0 is higher than public acceptance of FAST, the reason for this being that Stroke 1‐2‐0 is easier to remember (Zhao et al., 2020). The relatively higher level of awareness respondents in the present study showed of Stroke 1‐2‐0 in comparison with FAST indirectly reflects this preference for Stroke 1‐2‐0 as a stroke educational tool among older residents in the community.

Television was commonly reported as an important source for acquiring health‐related knowledge, yet it was not related to awareness of stroke educational tools in this study. This indicates that older adults may not be interested in watching advertisements that introduce stroke‐related knowledge. Therefore, promoting stroke knowledge using Wechat or a community bulletin board may be more effective than using television. Using these mediums could also reduce the financial burden of a mass media stroke campaign, as promoting stroke knowledge using television advertisements is costly. With the development of general practice in recent years, each community in China has been provided with a family physician team. In the future, stroke physicians should collaborate with family physicians to promote stroke knowledge in these promising and cost‐effective ways.

## 5 LIMITATIONS

We must admit that this study has its limitations. First, this was a voluntary questionnaire study. Although the response rate was more than 90%, there may have been differences between the knowledge of stroke of those excluded from the study and those who were included. Second, we only included two suburban communities in this study that may not be representative of all the suburban areas in Shanghai. A large cross‐sectional survey that includes more communities in different regions across China should be conducted in the future. Third, the awareness rate of stroke symptoms and risk factors among older adults may have been overestimated because of the use of close‐ended questions. A previous study compared open‐ended with close‐ended questions regarding the awareness of stroke symptoms (Rowe et al., 2001), and it showed that when participants were given an open‐ended question, less than 40% could name at least one of the five stroke symptoms included in SUDDEN, whereas none of them were able to name all five. However, when warning signs were read from a list, almost all of them identified at least one symptom, and more than half identified all five. This indicates that the results vary significantly depending on the way questions are asked. Therefore, as with the results of many previous studies, our results regarding the awareness of stroke symptoms and risk factors should be interpreted with caution. Nevertheless, the awareness of stroke educational tools as indicated by our study should not be affected by the way questions were asked, as we asked further questions regarding the meanings of Stroke 1‐2‐0 and FAST.

## 6 CONCLUSION

Stroke 1‐2‐0 and FAST are two promising tools that might enable members of the public to recognize the onset of stroke and act immediately. The limited awareness of these tools among older adults in the community indicates that we need to take action to improve awareness of them. Promoting stroke knowledge using Stroke 1‐2‐0 is recommended in China, and a regular review of the effectiveness of promoting such tools is also necessary.

## AUTHOR CONTRIBUTIONS

*Study concept, analysis and interpretation of data, and drafting the manuscript*: Ling Ling. *Study concept, acquisition of data, analysis and interpretation of data, and drafting the manuscript*: Zhongcheng Li. *Acquisition of data and fundraising*: Sichen Yao. *Study concept and design, study supervision and coordination, and revising the manuscript*: Xiaochuan Liu. *Study concept and design, study supervision and coordination, revising the manuscript, and fundraising*: Jing Zhao. *Contributed equally to this work*: Ling Ling and Zhongcheng Li.

### PEER REVIEW

The peer review history for this article is available at https://publons.com/publon/10.1002/brb3.2357

## CONFLICT OF INTEREST

The authors declare no conflict of interest.

## Supporting information

Supporting Information

## Data Availability

All data produced by this study are obtainable if the request is reasonable. Please contact the corresponding author.
