# Supplementary material for: Poor awareness of stroke educational tools among older adults in China
Source: Brain Behav. 2021 Sep 14;11(10):e2357. doi: 10.1002/brb3.2357 (PMC8553324; doi:10.1002/brb3.2357)
Supplement: Supplementary file 1 — Supporting Information [file BRB3-11-e2357-s001.docx]

**Online supplemental material**

Stroke awareness survey in community-living older adults

Part 1 Basic information

1. Your name:
2. Room number:
3. Gender:
4. Age:
5. Educational background: primary school (<6 years)

secondary school (6-9 years)

high school (9-12 years)

college degree or higher

Part 2 Past medical history

1. Smoking history: quit smoking for more than one year

never quit

never smoke

1. Alcohol consumption: quit drinking for more than one year

never quit

never drink

1. Hypertension: Yes/No
2. Diabetes: Yes/No
3. Prior Myocardial Infarction: Yes/No
4. Prior Stroke: Yes/No
5. Dyslipidemia: Yes/No
6. Atrial fibrillation: Yes/No
7. Cancer history: Yes/No

(Smoking and drinking habit were self-reported. All the medical history was confirmed with the medical record from local tertiary hospitals.)

Part 3 Stroke awareness knowledge

1. Choose the symptoms listed below which you think are indicative to the attack of stroke? (multiple choice)

Sudden trouble seeing in one or both eyes (Yes/No)

Sudden, severe headache with no known cause. (Yes/No)

Sudden dizziness, loss of balance or coordination (Yes/No)

Sudden trouble speaking or understanding (Yes/No)

Sudden numbness or weakness of the arm or leg (Yes/No)

Sudden face drop (Yes/No)

1. Choose the factors listed below which you think are risk factors for stroke? (multiple choice)

Hypertension (Yes/No)

Diabetes (Yes/No)

Smoking (Yes/No)

Alcohol abuse (Yes/No)

Lack of exercise (Yes/No)

Dyslipidemia (Yes/No)

Heart disease (myocardial infarction, atrial fibrillation, etc) (Yes/No)

Obese (Yes/No)

1. Have you ever heard about any of the therapy for ischemic stroke?

thrombolytic therapy

thrombectomy therapy

both of them

neither of them

1. Will you call the emergency medical service once you spot a stroke at home?

Yes, I will.

No, I won’t. **(please give the reason)**

1. Have you ever heard about any of the stroke awareness tool for stroke education?

FAST (Face, Arm, Speech, Time)

Stroke 1-2-0

Neither of them

Both of them

1. Where do you commonly get the stroke-related knowledge in daily life? (multiple choice)

Television

Wechat

Community bulletin board

Others **(please introduce some ways)**

(Wechat is a popular social communication application in China, people share diverse information with each other using this application.)

1. For responders who have heard the stroke awareness tool FAST, please explain the meaning of each letter.

Know the meanings of FAST

Know part of the meanings of FAST

Can’t name any of the meanings of FAST

1. For responders who have heard the stroke awareness tool Stroke 1-2-0, please explain the meaning of each number.

Know the meanings of Stroke 1-2-0

Know part of the meanings of Stroke 1-2-0

Can’t name any of the meanings of Stroke 1-2-0
